# Evaluation of self-collection of nasal swab specimens for COVID-19 diagnostic testing in children in the United States, 2020–2023

**DOI:** 10.1128/jcm.01336-25

**Published:** 2026-03-30

**Authors:** Zachary R. Smith, Andrew Godoshian, Peter Boersma, Christy Myrick, Ellen N. Kersh, Rebecca J. McNall, Erika Mey, Akilah R. Ali, Hannah Allen, Gregg Tavolacci, Coral May, Dean L. Winslow, Michael F. Iademarco, Marcus Siddall, Joseph Miller

**Affiliations:** 1 Office of Readiness and Response, Centers for Disease Control and Prevention 1242, Atlanta, Georgia, USA; 2 National Center for Health Statistics, Centers for Disease Control and Prevention 37345, Hyattsville, Maryland, USA; 3 Office of Laboratory Systems and Response, Centers for Disease Control and Prevention 1242, Atlanta, Georgia, USA; 4 University of California-Irvine 8788, Irvine, California, USA; 5 eTrueNorth, Mansfield, Texas, USA; 6 Stanford University School of Medicine 10624 , Stanford, California, USA; 7 U.S. Public Health Service, Washington, DC, USA; Wadsworth Center - NYSDOH, Albany, New York, USA

**Keywords:** diagnostics, specimen collection, SARS-CoV-2

## Abstract

The COVID-19 pandemic highlighted a critical need for scalable and efficient diagnostic testing strategies to contain the spread of SARS-CoV-2, particularly among children. We evaluated the reliability of self-specimen collection in children aged 5–17 years by analyzing two large data sets. First, we examined 78,844 rapid antigen tests conducted at federal sites from 26 May 2023 to 31 January 2024, comparing rates of positive test results among unaccompanied children with a median age of 16. A generalized linear mixed model accounting for site-level variation found no significant difference associated with self-collection versus healthcare worker (HCW) collection (odds ratio = 1.84, *P* = 0.212). Symptom status was the strongest predictor of a positive test result, with symptomatic children having over 40 times the odds of a positive result; among symptomatic children, specimen self-collection was associated with higher odds of a positive result. Second, we assessed RNase P internal sample control signal across 981,631 self-collected real-time reverse transcriptase polymerase chain reaction tests at community testing sites. RNase P detection failure rates were low across all age groups, and there were no significant differences among children aged 5–17, adults aged 18–64, and older adults. Together, these findings demonstrate that self-specimen collection by children yields diagnostically adequate and reliable results, comparable to HCW collection and adult self-collection during a pandemic response.

**IMPORTANCE:**

This study provides robust evidence supporting the implementation of self-collection of specimens in children, which could significantly enhance testing accessibility and efficiency in public health responses.

## INTRODUCTION

The COVID-19 pandemic necessitated widespread and frequent diagnostic testing to monitor and contain the spread of SARS-CoV-2. While testing in children has traditionally relied on specimen collection by healthcare workers (HCWs), the pandemic’s scale and urgency prompted broader use of self-collected anterior nasal swabs. Self-specimen collection offers several advantages, including reducing the burden on healthcare systems for specimen collection, minimizing HCW exposure to infectious agents, lowering the level of personal protective equipment required for specimen collection, increasing patient convenience, decreasing test administration time, increasing testing throughput, and expanding test accessibility.

Several studies have already demonstrated that self-administered anterior nasal specimen collection has reasonable accuracy and sensitivity (1, 2), and there was high agreement between self-collected and HCW-collected specimens (3, 4) for detecting SARS-CoV-2. In addition, classroom-based studies (4, 5) have shown that children as young as kindergarten age can successfully self-collect nasal swabs under adult supervision, as well as achieve high rates of adequate sampling based on RNase P detection (6). Despite providing valuable insights, these prior studies were limited in scale, methodology, and public health relevance. To address this, we leveraged the CDC Increasing Community Access to Testing, Treatment, and Response (ICATT) program to evaluate the feasibility and reliability of pediatric self-collection at scale and in varied environments from two complementary retrospective studies, under pandemic conditions.

During the COVID-19 pandemic, the ICATT program performed 49.2 million diagnostic tests at federal sites, community pharmacies, and community surge sites (7, 8). This study compared the efficacy of self-specimen collection versus HCW specimen collection in children aged 5–17 years at federal facilities that exclusively utilized either method. Second, we examined RNase P cycle threshold (Ct) values, a surrogate marker of specimen quality, to compare self-specimen collection in children and adults. Together, these analyses add to prior controlled studies (4–6) by evaluating pediatric self-collection in high-throughput, real-world settings, highlighting the value of using the ICATT network and testing targets to evaluate testing recommendations and policies (8).

## MATERIALS AND METHODS

### Study design and data processing

This study consisted of two complementary retrospective analyses conducted using data from the CDC ICATT program. Both analyses focused on pediatric self-collection of anterior nares swabs for SARS-CoV-2 testing, using both point-of-care antigen and laboratory-based real-time reverse transcriptase polymerase chain reaction (RT-PCR) diagnostic tests.

The first study evaluated COVID-19 rapid antigen test data collected from multiple U.S. Customs and Border Protection sector sites between 26 May 2023 and 31 January 2024 to compare the effectiveness of self-specimen collection versus HCW-assisted specimen collection in children. The data set included age, sex, symptom status, testing date, test result, specimen collection method (self versus HCW), site location, and regional COVID-19 incidence rate. Incidence estimates were based on weekly cases per 100,000 people in the neighboring Mexican states of Tamaulipas, Coahuila, Chihuahua, Sonora, and Baja California as most children were recent arrivals from those regions. To ensure spatiotemporal consistency in the study population, we restricted the analysis to children aged 5–17 years, excluded tests conducted outside of the date range, and excluded records where symptom status was not reported.

Children were tested for SARS-CoV-2 infection via a rapid antigen test (BinaxNow; Abbott, Lake Forest, IL) provided through ICATT partner eTrueNorth (Mansfield, TX). Each of the seven federal sites exclusively used either self-specimen collection or HCW specimen collection. At sites in Rio Grande Valley (TX) and El Paso (TX) sectors, children self-collected specimens, while at sites in Laredo (TX), Del Rio (TX), Tucson (AZ), Yuma (AZ), and San Diego (CA), healthcare workers collected specimens following the test’s instructions for use (IFU) (9). Children who self-collected specimens were provided directions verbally in Spanish and/or were provided a demonstration on a nose model or mock demonstration by a healthcare worker. Following the child’s swabbing of their anterior nares, the remainder of the COVID-19 testing procedure was conducted by an HCW in accordance with the IFU.

To complement the first analysis and to further assess pediatric self-specimen collection, we conducted a second analysis focused on specimen adequacy using RT-PCR detection of the RNase P gene, a human RNA internal sample control. We evaluated 981,631 self-collected nasal swab tests conducted at 1,243 pharmacy and surge sites from 19 October 2020 to 24 June 2023 using the TaqPath COVID-19 Combo Kit (Thermo Fisher Scientific, Waltham, MA) (10). RNase P was used as a surrogate marker for swabbing quality, with a Ct value of ≤33 indicating an adequate sample and a Ct of >33 indicating an insufficient sample (10). Participants were grouped by age: 5–17, 18–64, and ≥65 years. Prior to 19 October 2020, self-collection was not authorized by the U.S. FDA for this assay (11). While all specimens in this data set were designated self-collected, assistance may have been provided in some cases when necessary. Samples outside the stability time range or leaking were not tested.

This activity was reviewed by the CDC, deemed not research, and therefore did not require IRB review. All research was conducted consistent with applicable federal law and CDC policy (12). All diagnostic testing was performed by eTrueNorth.

### Statistical analysis

Because no federal site used both specimen collection methods on children aged 5–17 years, we employed a generalized linear mixed model (GLMM) with site as a random intercept that allowed us to estimate the effect of specimen collection type while accounting for site-level differences.

The primary outcome for the self- or HCW-specimen collection analysis was binary SARS-CoV-2 test result (0 = negative, 1 = positive). Covariates included symptom status (symptomatic or asymptomatic), sex (male or female), age in years, community rate of positive test results (regional weekly cases per 100,000 population), and testing date (converted to a numeric variable). In the GLMM, specimen collection method was modeled as a fixed effect and site as a random intercept. To evaluate whether including site as a random effect improved model fit over the naïve logistic approach, we compared log-likelihood values and performed likelihood ratio tests. In addition, we performed a stratified analysis by symptom status to determine whether the relationship between specimen collection method and rate of positive test result varied between symptomatic and asymptomatic children.

To evaluate children self-collection versus adult self-collection in the community testing data set, we used logistic regression models with RNase P detection failure as the outcome. Predictors included age group (5–17, 18–64, and ≥65 years), sex, and SARS-CoV-2 variant wave. We also included days from collection to laboratory receipt of specimens and days from receipt to test result, both of which were standardized prior to modeling.

All models were fit using maximum likelihood estimation with PySpark for data preprocessing and model fitting using the statsmodels.formula interface in Python (v3.9).

## RESULTS

### Children self versus HCW specimen collection using antigen tests

**Fig 1:**
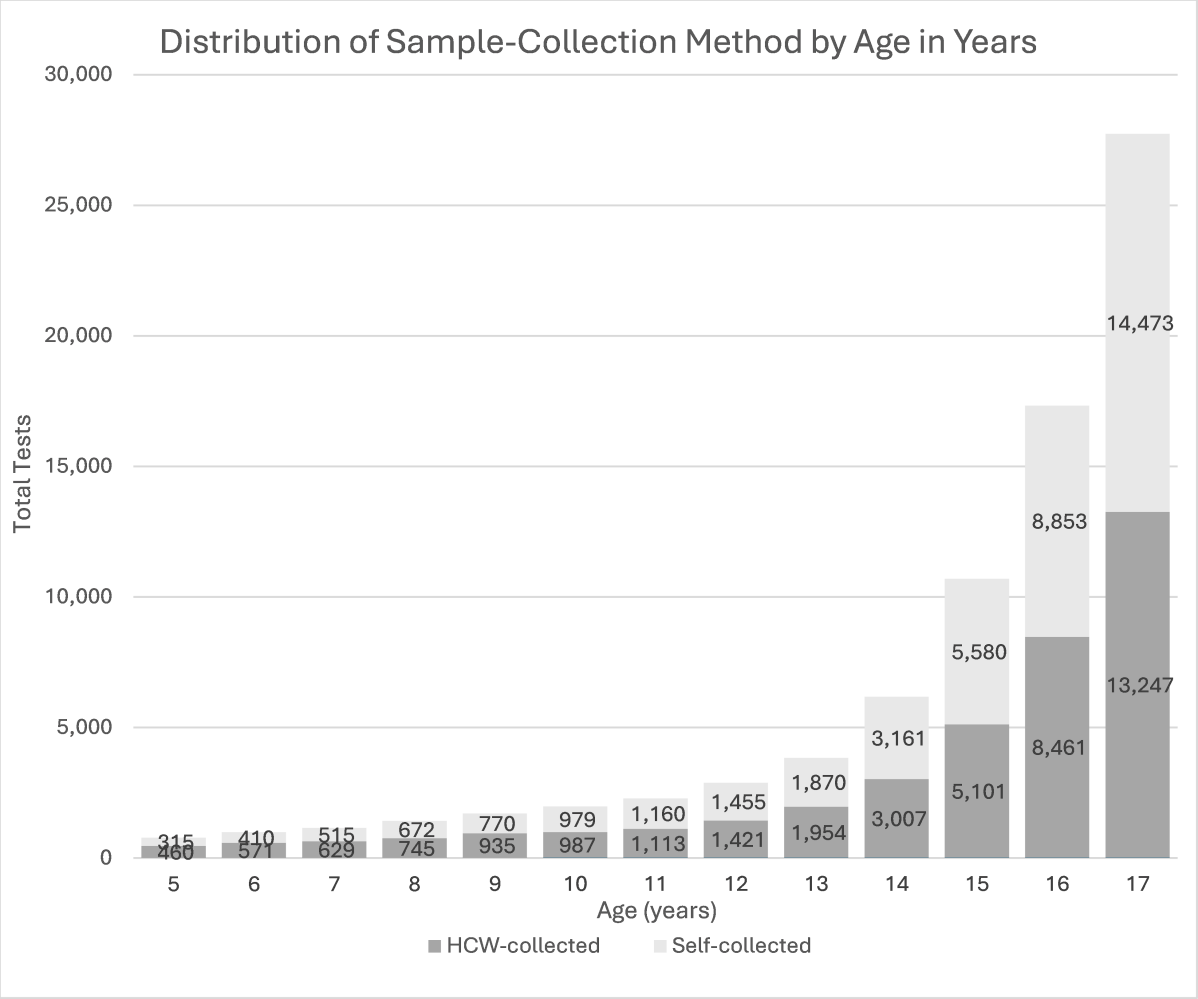
Children tested using point-of-care antigen devices at seven federal sites from 26 May 2023 to 31 January 2024. Light gray bars depict self-collected specimens, and dark gray bars depict healthcare worker (HCW)-collected specimens.

A total of 78,844 point-of-care antigen tests were performed across the seven federal sites (Table 1). Overall, 63% were boys and 37% were girls, each with a median of 16 years of age. Specimen collection method was roughly split evenly among children of every age (Fig. 1). Among all children, 4.9% self-reported symptoms and 3.2% had positive test results for SARS-CoV-2. Self-specimen collection was conducted at locations in Rio Grande Valley and El Paso sectors, accounting for 51% of all tests, whereas HCW specimen collection comprised the remainder (49%) collectively in Laredo, Del Rio, Tucson, Yuma, and San Diego sectors.

**TABLE 1:** Patient demographics and antigen test positivity by federal site

	Self-collected specimen	Male (%)	Female (%)	Median age	Tests	Positive (rate, %)
Rio Grande Valley (TX)	Yes	60	40	16	27,979	1,637 (5.9)
El Paso (TX)	Yes	66	34	16	12,234	199 (1.6)
Laredo (TX)	No	70	30	16	1,889	57 (3.0)
Del Rio (TX)	No	60	40	16	12,307	250 (2.0)
Tucson (AZ)	No	62	38	16	21,550	319 (1.5)
Yuma (AZ)	No	61	39	16	1,678	43 (2.6)
San Diego (CA)	No	65	35	16	1,207	12 (1.0)

**Fig 2:**
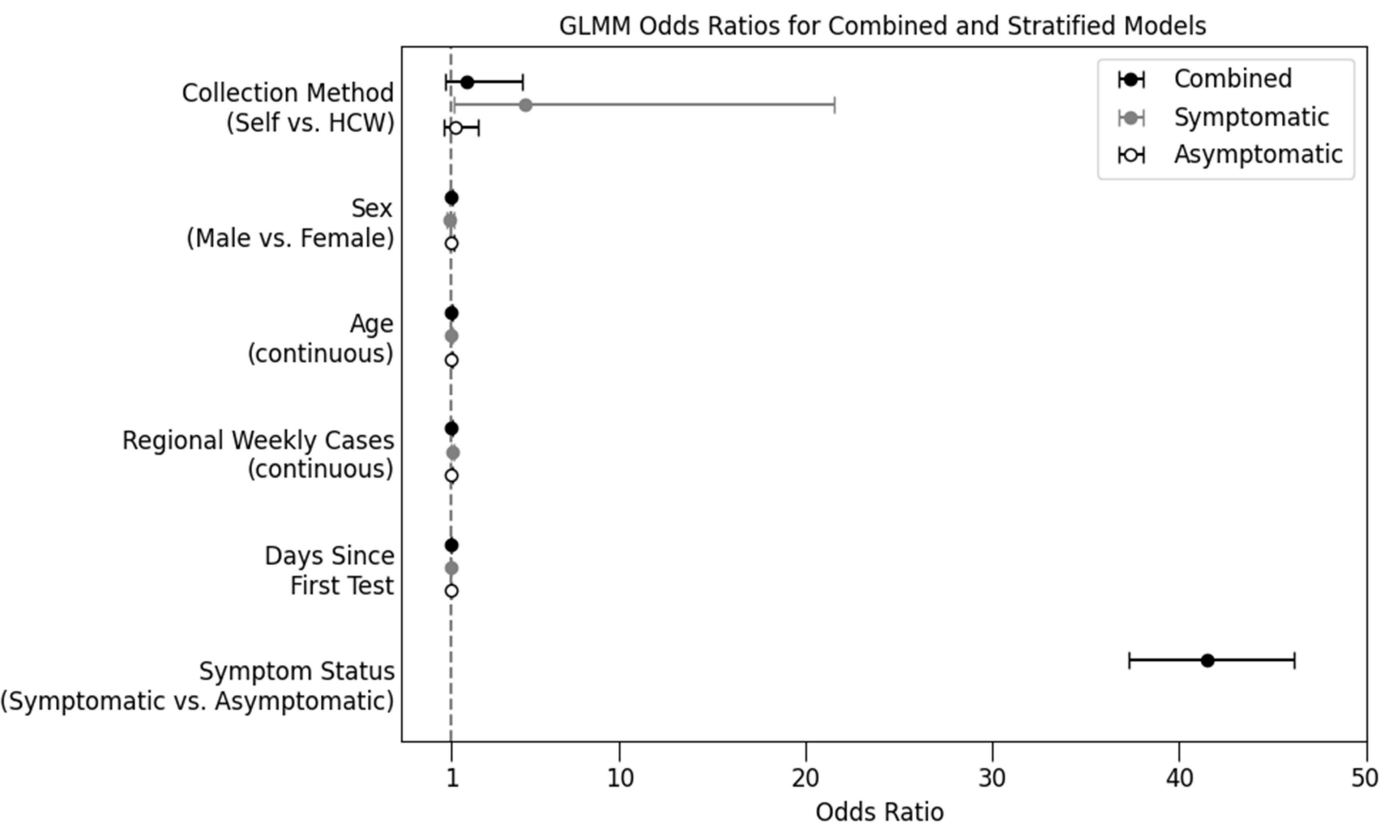
Odds of a positive test result in relation to covariates in a generalized linear mixed model incorporating federal site as a random intercept. Whiskers delimit 95% confidence intervals. Overall, self-specimen collection in children tested by point-of-care antigen tests was not significantly different from healthcare worker (HCW) collection (OR = 1.84, CI: 0.71–4.77, *P* = 0.212). In a stratified analysis, specimen self-collection was significantly more likely to yield a positive result among symptomatic children (OR = 4.95, CI: 1.14–21.48, *P* = 0.033).

The odds ratio (OR) of positive test results by self-specimen collection was 1.84 (95% confidence interval [CI]: 0.71–4.77, *P* = 0.212), indicating no significant difference between self-collection and HCW collection in children (Fig. 2). Presence of symptoms remained the most significant predictor, with symptomatic children having over 40 times greater odds of a positive test result than asymptomatic children (41.5; 95% CI: 37.3–46.2, *P* < 0.001). Neither age, sex, pandemic wave, nor regional weekly case rate was statistically significant.

When stratifying by symptom status, self-specimen collection was significantly associated with a positive test outcome for symptomatic children (OR = 4.95, *P* < 0.033) but not for asymptomatic children (OR = 1.237, *P* = 0.543). Regional incidence rate was weakly predictive only for symptomatic children (OR = 2.352, *P* = 0.019, for symptomatic; OR = 1.00, *P* = 0.886, for asymptomatic). In addition, there was a slight but significant decrease in the rate of positive test results over time for asymptomatic children (OR = 0.995 per day, *P* < 0.001).

### Specimen collection proficiency by age using RT-PCR tests

A total of 981,631 individuals were tested via self-collected nasal swabs at pharmacy and surge testing sites between 19 October 2020 and 24 June 2023. Of these, 11.3% were children aged 5–17 years; 78.1% were adults aged 18–64 years; and 10.6% were aged 65 years and older. The median age was 37 years, and 57.3% of the patients were female.

**Fig 3:**
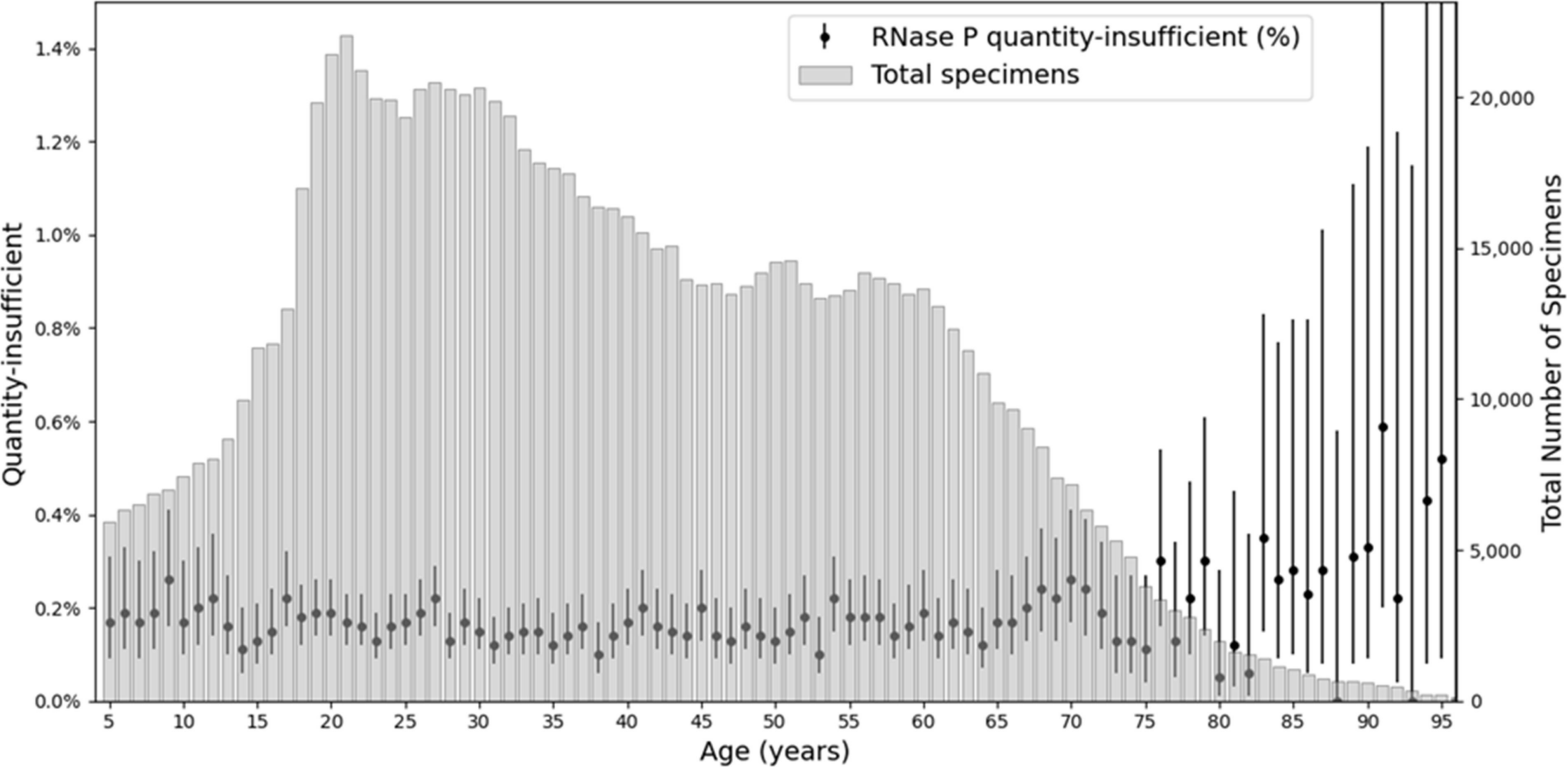
Age-specific proportion of RNase P quantity-insufficient specimens and total specimen counts by age: percentage of specimens at each age with RNase P quantity-insufficient results. Whiskers denote 95% confidence intervals (black) and total number of specimens tested at each age (gray). Quantity-insufficient rates were low and not significantly different across the age of patients.

The overall RNase P detection failure rate was 0.16%, indicating a high level of proper specimen collection. Among children aged 5–17 years, the failure rate was 0.18% (Fig. 3). In adults aged 18–64, the failure rate was 0.16%, while it was highest among individuals aged 65 years and older at 0.20%.

**Fig 4:**
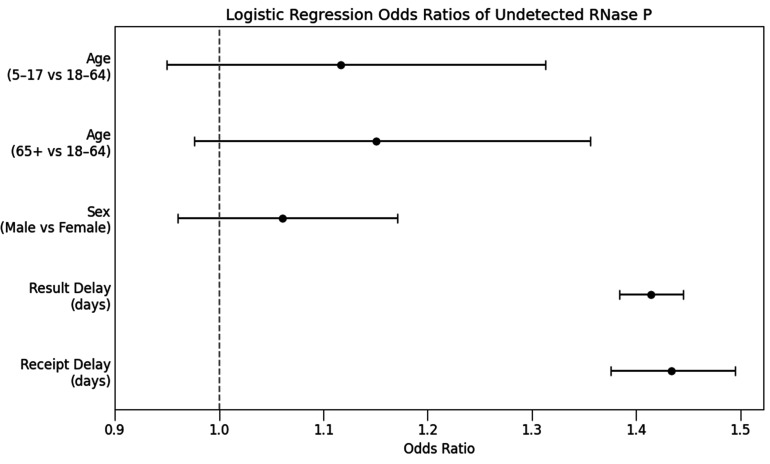
Odds of failure to detect RNase P in 981,631 samples from pharmacy and surge sites in relation to covariates in a logistic regression model. Whiskers delimit 95% confidence intervals. Children aged 5–17 years did not have significantly higher odds of quantity not sufficient (QNS) results compared to adults aged 18–64 years, nor did those aged 65 and over. Delays in specimen transport and processing were the strongest predictors of QNS.

A logistic regression model (Fig. 4) assessing predictors of RNase *P* detection failure found no statistically significant difference between children (OR = 1.12, 95% CI: 0.950–1.313, *P* = 0.181) or adults aged 65+ (OR = 1.150, 95% CI: 0.976–1.356, *P* = 0.095) relative to adults aged 18–64.

Delays in specimen handling were the strongest predictors of quantity not sufficient (QNS) results. Each day of delay between specimen collection and laboratory receipt increased the odds of RNase P detection failure by 43.4% (OR = 1.434, 95% CI: 1.376–1.494, *P* < 0.0001). Delay from receipt to result also significantly predicted QNS results (OR = 1.414, 95% CI: 1.384–1.445, *P* < 0.0001). The association between processing delay and QNS was significantly stronger among adults aged 65 and older (OR = 1.165, 95% CI: 1.089–1.246, *P* < 0.0001) compared to adults aged 18–64.

## DISCUSSION

These large, real-world studies provide robust evidence that self-specimen collection is a reliable method for detecting SARS-CoV-2 in children aged 5–17 years. First, analysis of rapid antigen testing with exclusive use of either self- or HCW-collected specimens indicates that children proficiently self-collect specimens. Second, evaluation of sample quality using RT-PCR detection of RNase P shows that children self-collect specimens with suitable diagnostic yield.

The odds ratios from the GLMM accounting for site-level variation indicate that self-collection of specimens by children was not inferior to HCW collection. If anything, for symptomatic children, self-specimen collection was associated with higher odds of detection over HCW collection. These findings align with prior smaller studies that support comparable performance between self- and HCW-collected anterior nasal swabs among children (4), similar accuracy and sensitivity for self-administered BinaxNOW antigen tests (13), and high concordance in repeated high-demand settings (1). Meta-analyses have similarly reported strong agreement between self- and HCW-administered testing protocols (14, 15).

Rio Grande Valley sector (using only self-collection) had the highest rate of positive test results, whereas the Tucson sector (using HCW collection) had the lowest. The GLMM, which adjusted for these site effects, showed no decrement in performance of self-collection and confirmed a significant association for symptomatic children. These results suggest that while local factors may affect absolute rate of positive test results, self-collection remains diagnostically reliable across heterogeneous field conditions. The strong association between symptom status and rate of positive test results also confirms previous observations that symptomatic individuals carry higher viral loads and are more readily detected by both rapid antigen and molecular assay (16, 17).

The second component of our study reinforces this conclusion from an independent angle: adequacy of specimen self-collection measured via RNase P detection across a much larger, generalizable cohort. Failure rates for RNase P detection were low overall and were not significantly different among children compared to adults. The odds of QNS in children aged 5–17 years were not significantly different from adults aged 18–64 years. These findings extend earlier work demonstrating that children can collect high-quality specimens under supervision (6) in scalable, decentralized, real-world settings.

Together, these findings support the conclusion that self-specimen collection is feasible, reliable, and diagnostically sound in children, even outside clinical environments. The strong association between symptom status and rate of positive test results further reinforces the importance of timely and accessible testing. Given the benefits, including reduced strain on healthcare resources, lower labor costs, and increased accessibility, implementation of self-specimen collection in similar high-demand settings should be prioritized regardless of age group.

There are limitations. Due to the lack of overlap between the specimen collection method and the site location, these cannot be fully disentangled. Any estimated effect of specimen collection method may be partially attributable to unmeasured site-level characteristics not accommodated by the GLMM approach. Symptom status was based on self-report or disclosure to HCWs and may have been underreported by children due to fear of adverse consequences. Although instructions were provided in Spanish and often accompanied by demonstrations, language and communication barriers could have influenced technique. While federal sites had consistent protocols during the study period, some sites transitioned from self- to HCW collection over time, possibly introducing procedural drift. At community testing sites and the two federal sites where self-collection of specimens was the default protocol, unrecorded exceptions for children or adults requiring assistance may have occurred; however, these were believed to be rare based on observation. Although RNase P detection indicates that human nucleic acid was successfully collected, isolated, and amplified, this result indirectly indicates the collection of an adequate specimen for the detection of SARS-CoV-2. In addition, because of the sparse distribution of the 1,571 QNS results across the 1,243 testing sites studied, estimation of any site effect was not possible.

In sum, both the federal and community site analyses converge to support the use of self-specimen collection for SARS-CoV-2 testing in school-aged children. Given its advantages of reducing burden on healthcare workers, decreasing PPE requirements, increasing operational efficiency, and greater scalability, self-collection could be considered a reliable and effective strategy for expanding access to diagnostic testing among children in congregate settings.

## Data Availability

Anonymized line-level data are available on request to the corresponding author, subject to a CDC-approved data use agreement.
